# Adult-Brain-Derived Neural Stem Cells Grafting into a Vein Bridge Increases Postlesional Recovery and Regeneration in a Peripheral Nerve of Adult Pig

**DOI:** 10.1155/2012/128732

**Published:** 2012-02-02

**Authors:** Olivier Liard, Stéphanie Segura, Emmanuel Sagui, André Nau, Aurélie Pascual, Melissa Cambon, Jean-Luc Darlix, Thierry Fusai, Emmanuel Moyse

**Affiliations:** ^1^Unité de Chirurgie et Physiologie Expérimentale (UCPE), Institut de Médecine Tropicale, 58 boulevard Charles Livon, 13007 Marseille, France; ^2^Unité Physiologie de la Reproduction et des Comportements, UMR 85, Centre INRA de Tours, 37380 Nouzilly, France; ^3^Laboratoire d'Ecologie fonctionnelle, Bâtiment 4R3, 118 route de Narbonne, 31062 Toulouse cedex 9, France; ^4^Unité de Rétrovirologie, U421 INSERM, Ecole Normale Supérieure de Lyon, 46 Allée d'Italie, 69364 Lyon cedex 07, France

## Abstract

We attempted transplantation of adult neural stem cells (ANSCs) inside an autologous venous graft following surgical transsection of *nervis cruralis* with 30 mm long gap in adult pig. The transplanted cell suspension was a primary culture of neurospheres from adult pig subventricular zone (SVZ) which had been labeled *in vitro* with BrdU or lentivirally transferred fluorescent protein. Lesion-induced loss of leg extension on the thigh became definitive in controls but was reversed by 45–90 days after neurosphere-filled vein grafting. Electromyography showed stimulodetection recovery in neurosphere-transplanted pigs but not in controls. Postmortem immunohistochemistry revealed neurosphere-derived cells that survived inside the venous graft from 10 to 240 post-lesion days and all displayed a neuronal phenotype. Newly formed neurons were distributed inside the venous graft along the severed nerve longitudinal axis. Moreover, ANSC transplantation increased CNPase expression, indicating activation of intrinsic Schwann cells. Thus ANSC transplantation inside an autologous venous graft provides an efficient repair strategy.

## 1. Introduction

Nerve injuries which are frequent in civil practice [1, 2] and catastrophe-like earthquake [3, 4] or tsunami [5] combine associative lesion with crush syndrome. War improves the risk of gap and of treatment delay with ballistic wounds [6] because these ones are less prone to emergency than vascular injuries [7]. This risk pertains after war with remanent explosive [8]. The functional pronostic is dependent on the type of wound: transsection, crush, or gap, which has been further categorized according to the length of the gap, the type of nerve (sensitive, motor or mixed), localization, association of injuries, and patient's ageing, time of surgery, and vascular environment [9–12].

Neuroguides and especially vein grafts are regularly used to bridge small nerve gaps shorter than 3 cm; for long nerve gaps, autologous nerve graft has become the gold standard since the second war [13, 14]. But nerve repair outcome depends on fascicle concordance [15] and scar development, so expected alternatives for long nerve gaps remain a challenge to improve recovery after this type of lesion. Surgical trials to improve nerve repair have mainly targeted diversification of grafted neuroguides with resorbable materials [16]. The outcome has been improved by neuroguide preloading with extracellular matrix components [17–19], growth factors [20], or cultured regeneration-enhancing cells. The latter strategy, or cell therapy, has been attempted with primary cultures of Schwann cells [21, 22], olfactory bulb enshealting cells [23], or various types of stem cells [24]. In these studies, progenies of grafted stem cells mostly proved to be glial cells, which indirectly enhance axonal regeneration [24].

Neural stem cells from adult brain (ANSC) have been scarcely assayed to help postlesional repair of peripheral nerves, despite their strong potential of integration and development into neurons [25]. The present study is based on the hypothesis that ANSC grafting inside a venous neuroguide might be efficient to bridge a long nerve gap in adult. We tested this hypothesis in the pig, which is the non-primate animal species most closely related to human and is prone to surgery and anesthesia conditions very close to human protocols in clinic [26]. To this aim, we have characterized primary cultures of neural stem cells from adult pig brain, by optimizing the neurosphere assay on subventricular zone explants [27]. In the present study, we labeled such primary expanded ANSC *in vitro* with either BrdU or lentiviral green fluorescent protein gene transfer, and we grafted labeled neurospheres into an autologous venous bridge after a surgical 30 mm long gap in adult pig *nervi cruralis*. We compared neurosphere-grafted and control pigs for functional recovery and assessed the fate of grafted cells inside the lesioned nerve at various post-lesion intervals. 

## 2. Material and Methods

### 2.1. Animals

Twenty-four adults, 4-month-old Large White Landrace pigs were used in the present study. They were housed with *ad libitum* access to water and standard food pellets, in lodges that comprised one yard and one hard infrared-heated shelter. All *in vivo *experimental protocols were approved by the local Ethics Committee on the protection of animals used for scientific activities with the protocol number 31/2006/IMTSSA/UCPE. Personal protocol number for surgery and experimentation with pigs was 2007/29/DEF/DCSSA (to O. Liard). All efforts were made to minimize the number of animals used and their suffering.

### 2.2. Anesthesia

Deep anesthesia was initiated by intramuscular premedication with 30 mg/kg ketamine (Imalgene 1000, Merial, Lyon, France), 0.1 mL/kg acepromazine (Vetranquil, Calmivet, Bayer, Puteaux, France), and 25 *μ*g/kg atropine sulphate (Meram, Melun, France). The animal was then equipped with a peripheral intravenous perfusion delivering Ringer-lactate-5% glucose serum at 5–7 mL/kg/h, with adhesive electrocardiographic electrodes in CM5-SpO2 (ECG monitor S5, Datex Ohmeda, Madison, WI, USA), and with an artificial oxygenator. Anesthesia induction was performed by intravenous injections of 2 mg/kg propofol (Diprivan, Bayer) during 30 sec slowly, and of sufentanil (Sufenta, Bayer) three times at 1.5 *μ*g/kg. Local glottis anesthesia was made with 5% lidocaïne spray (Xylocaine, Bayer), before orotracheal intubation with a monolight cannula (internal diameter 6.5 mm). Ventilation of lung parenchyma was checked with a stethoscope and mechanically assisted. General anesthesia was sustained with electrically driven intravenous infusion of propofol 2 mg/kg/h (Diprivan, DCI), sufentanil 1 *μ*g/kg/h (Sufenta, DCI) and rocuronium bromide 0.15 mg/kg/h (Esmeron, DCI); curarisation was supervised by means of a S5 monitor (Entropie module) but was not released for EMG. Antibioprophylaxy was administered. All along surgery, the cardioventilatory parameters (heart and ventilation rates, electrocardiogram, blood oxygenation, ventilation pressure and volume, MAC, temperature, hemoglucotest) as well as the sleep threshold were constantly watched with the S5 monitor.

### 2.3. Brain Tissue Sampling for Primary Neural Stem Cell Culture from Adult Pig

Separate animals were dedicated for primary culture and *in vitro* expansion of neural stem cells from brain subventricular zone (SVZ) as previously described [27]. On each deeply anesthetized pig, the dorsal face of the skull was cut with an electric saw and removed. A 15 mm thick coronal slice of the brain was performed with a scalpel at the commissural level of midbrain, manually explanted and transferred at 4°C on ice bed-laid sterile metal plate, beside a Bunsen flame for atmosphere sterilization. The experimental animal was thus alive until the explantation of the brain tissue slice. Each pig was sacrificed immediately after brain tissue sampling by intravenous injection of Dolethal (R) pentobarbital (DCI) at 1.5 mL/kg.

### 2.4. Primary Culture of SVZ Neural Stem Cells (“Neurosphere Assay”)

As previously described [27], SVZ pieces were quickly microdissected from the thick midbrain slice (above) along the ventrolateral floor of lateral brain ventricles in low-calcium artificial cerebrospinal fluid (aCSF: 124 mM NaCl, 5 mM KCl, 3.2 mM MgCl_2_, 0.1 mM CaCl_2_, 26 mM NaHCO_3_, 100 mM glucose, and pH 7.38). Tissue samples were rinsed twice with aCSF and digested first in 40 U cystein-EDTA-*β*-mercaptoethanol-preactivated papain (Sigma, L'Isle D'Abeau, France) for 10 min at 37°C, then in 250 *μ*L undiluted TrypLE Express solution (heat-resistant, microbially produced, purified trypsinlike enzyme, Gibco # 12604-013, Invitrogen, Cergy-Pontoise, France) for 10 min at 37°C. After the addition of 750 *μ*L of fresh aCSF and centrifugation for 8 min at 400 g at room temperature, the cell pellet was resuspended in 1 mL culture medium (DMEM (Sigma), 1x B27 (Gibco Invitrogen), 200 U/mL penicillin, and 200 *μ*g/mL streptomycin (Gibco Invitrogen)) containing 20 ng/mL Epidermal Growth Factor (EGF, Gibco Invitrogen) and 20 ng/mL basic Fibroblast Growth Factor (bFGF, Gibco Invitrogen). The cells were dissociated gently with a 26 G steel needle on a sterile disposable 1 mL syringe, counted on a Malassez slide, and seeded at 20,000 cells per mL in 6-well plates (Falcon, BD Biosciences, Bedford, USA). Cultures were monitored daily to follow the morphological growth of the neurospheres; passage was performed when the majority of spheres were 100–120 *μ*m in diameter. For passage, the primary spheres were collected in sterile tubes, incubated for 45–60 min at 37°C in 250 *μ*L undiluted TrypLE Express solution (Gibco Invitrogen) per 6 mL culture-derived pellet, and dissociated gently with a 26 G steel needle on a 1 mL sterile syringe; dispersed cells were centrifuged, counted, and seeded as above. All culture media were renewed by the replacement of 2 mL of medium per 4 mL well every 2-3 days. For immunocytochemistry, coverslips bearing differentiated spheres were rinsed in PBS, fixed in 4% paraformaldehyde-containing PBS for 20 min at 4°C, and kept in PBS at 4°C until the assay.

### 2.5. *In Vitro* Labelling of Neurosphere Cells

In the first set of experiments, tertiary neurospheres were incubated with the DNA synthesis precursor bromo-2-deoxy-uridine at 30 *μ*M (BrdU, Sigma) from 2 DIV after passage up to 8–10 DIV, BrdU being added fresh daily from concentrated stock.

**Figure 1:**
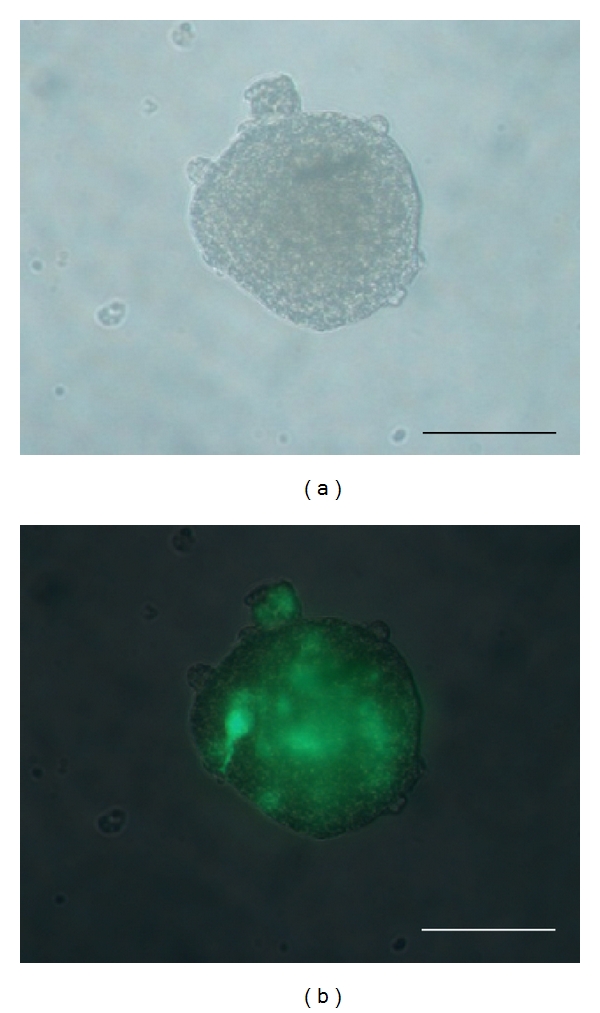
LV-GFP-labeled secondary neurosphere from adult pig SVZ, just before transplantation and 3 days after *in vitro* infection with the lentiviral vector of GFP, as observed under a photonic microscope with natural light (a) or GFP fluorescence (b). Scale bars: 50 *μ*m.

In a second set of experiments, lineage labelling was performed by infection of secondary neurospheres with a lentiviral vector of green fluorescent protein gene (LV-GFP) at 4-5 DIV after passage. LV-GFP was freshly synthetized, titrated, and tested as previously described [28], stored at −80°C and unfrozen just before use. Neurosphere cultures were preincubated 30 min in the presence of 1 mM polybrene (Sigma) and void lentiviral particles (VLP, 29) at 0.1x M.O.I., and further incubated 2 h with fresh culture medium containing 1 mM polybrene and LV-GFP at 0.3x M.O.I.; incubation medium was then replaced by fresh standard culture medium. LV-GFP-infected neurospheres were allowed 3 days culture in standard conditions for optimal lineage labelling (Figure 1); [28].

After optimal culture period (5-6 days for BrdU, 3 days for LV-GFP) and just before transplantation on the nerve lesion site (see below), neurospheres were collected, centrifuged 4 min at 800 g, and resuspended in fresh culture medium at 3 × 10^3^ spheres per mL.

### 2.6. Postlesional Grafting Surgery

The surgical approach was realized on the median face of thigh to isolate *nervis cruralis*. Nerve damage was established in the right *N. cruralis* by creating a 30 mm gap, which was then bridged with an autologous vein segment sampled from *v. mammelian externalis*. This venous neuroguide was sutured at both ends over the perinevre of the sectioned nerve. In some animals, once the venous shaft was sutured at the proximal end of the lesioned nerve, 300 *μ*L of freshly prepared neurosphere suspension (10^3^ spheres) were gently infused with a micropipet into the opposite end of the neuroguide, which was then sutured onto the distal lesioned nerve end. In other animals (controls), the venous neuroguide was sutured without any additional manipulation.

In the first set of experiments, lesioned pigs were thus grafted with autologous vein shafts which were either empty (controls, *N* = 2) or filled with BrdU-prelabeled neurospheres (*N* = 4). These animals were allowed 8 or 45 days survival after lesion.

In the second set of experiments, lesioned pigs were grafted with venous neuroguides which were either empty (controls, *N* = 3) or filled with LV-GFP-labeled neurospheres (*N* = 6). These animals were allowed 8 months survival after lesion.

### 2.7. Functional Analysis of Lesioned Animals

Animal behaviour was evaluated by assessing muscular atrophy, deficit of leg extension over the thigh. Each animal was examined before peripheral nerve lesioning and at days 45, 90, 180, and 240 following surgical bridging.

Motor function of the crural nerve was assayed by electromyography, using pregeled surface electrodes connected to a portable electromyographic recorder (Keypoint (R) v6.01, Medtronics, MN, USA). Bipolar concentric needles (diameter 0.47 mm) were applied on muscle *quadriceps femori* for stimulodetection analyses along *N. cruralis*. Stimuli were single 100 mA shocks, ranging from 0.1 to 1 msec durations. Electromyography was performed on animals before and at 45, 90, 180, and 240 days after transection. Values were recorded in terms of voltage, amplitude and time latency of detectable electromyographic activities. 

### 2.8. Immunohistochemistry

At the end of postlesional survival, the operated region of *N. cruralis* of each experimental pig was dissected out of the thigh under anesthesia and fixed by immersion in a 4% paraformaldehyde solution in 0.05 M, pH 7.4, NaH_2_PO_4_/Na_2_HPO_4_ buffer for 24 h at 4°C, then rinsed for 24 h at 4°C in 0.1 M, pH 7.4 phosphate-buffered saline (PBS). It was then cryoprotected for 72 h in a 30% sucrose solution, flat-embedded in OCT mounting medium and snap-frozen in liquid isopentane at −40°C for storage at −80°C. Longitudinal 20 *μ*m-thick sections were made in a cryostat (Leica 2800), mounted on commercial precoated slides (Superfrost-Plus, Fisher-Scientific, Canada), dried overnight at ambient air, and stored at −20°C.

For immunohistofluorescent labellings, these tissue sections were brought back to room temperature and permeabilized in 0.1 M PBS with 0.5% Triton-X-100. For nuclear antigens, tissue sections were also treated 15 min in 10 mM sodium citrate solution (pH 5.5) at 95°C. After a PBS rinse, all preparations were incubated in blocking buffer (0.1 M PBS, 0.1% Triton, 3% bovine serum albumin, 5% normal serum) for 1 h, then overnight at 4°C with one primary antibody (Table 1). After three 5-minute-rinses in fresh 0.1 M PBS, immunohistochemical labelings were revealed by 2 h incubation at room temperature in the dark with the appropriate Alexa-594-conjugated secondary antibodies (Molecular Probes, Eugene, OR, USA; 1/400). For double-staining experiments, Alexa-594-revealed slides were rinsed three times in fresh 0.1 M PBS and further incubated overnight at 4°C with another primary antibody, which was revealed with appropriate Alexa-488-conjugated secondary antibody (Molecular Probes; 1/200). After rinsing, fluorescent staining of cell nuclei was performed by 5 min incubation in the dark with DAPI (0.5 *μ*g/mL, Sigma). Labeled slides were coverslipped with aqueous mounting medium (Vectashield, Vector, Abcys, France) and photographed with a fluorescence microscope. CNPase immunohistochemical labeling was quantified on these photomicrographs by using the ImageJ software.

## 3. Results

### 3.1. Clinical Results

Before surgery, all experimental animals were healthy and displayed no walking deficit. After surgery, none of operated animals displayed any scar infection or host rejection response.

By clinical observation, loss of the right leg extension over the thigh was systematically observed after the surgical nerve transsection. This motor defect was maintained up to 240 days in animals which had crural nerve gap repaired by empty autologous venous bridge only. By contrast, lesioned leg extension over the thigh reappeared between 90 and 180 days in animals which had crural nerve gap repaired by the same type of autologous vein trunk filled with exogenous neurospheres.

### 3.2. Electromyography

Prior to surgery, electromyograms of m. quadriceps vast internal displayed amplitudes of 4.9 to 6.1 mV and poststimulus latencies of 0.89 to 3.7 ms. Immediately after surgical realization of *N. cruralis* substance loss, electromyograms of m. quadriceps were negative for all experimental animals, whatever their venous bridge had been filled or not with neurosphere suspension.

**Figure 2:**
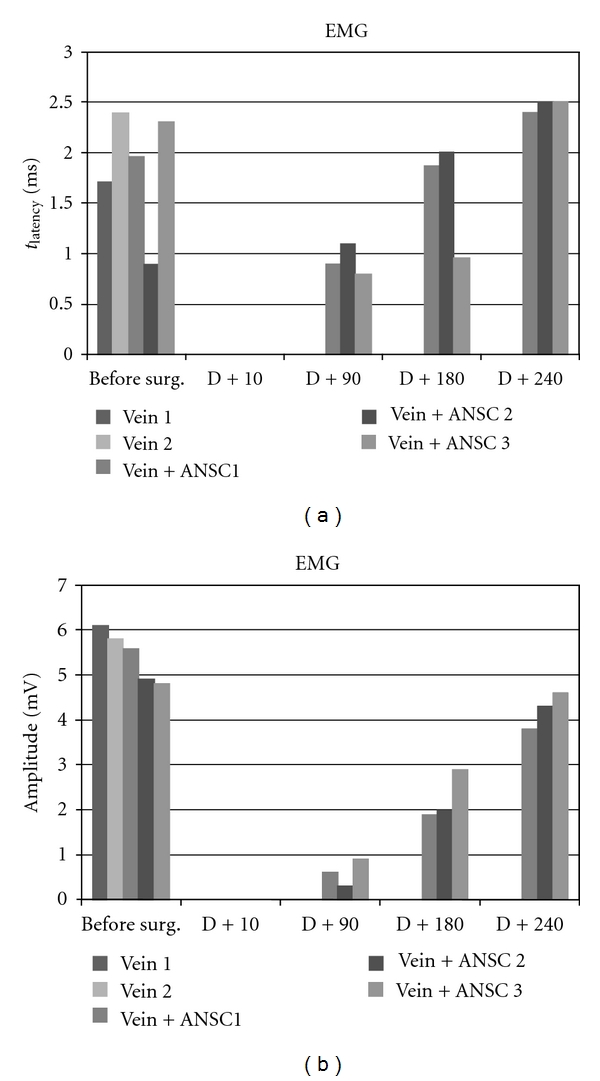
Electromyographic evaluation of muscle quadriceps femori at 90, 180, and 240 days following *N. cruralis* lesion and graft of GFP-labeled neurospheres inside a venous bridge before (“before surg.”) or after (“D + 90”, “D + 180”, “D + 240”) surgery.

At 180 days (25 weeks) after lesion, neurosphere-transplanted pigs displayed positive electromyograms of m. quadriceps with 0.6 to 0.9 mV amplitudes and 0.96 to 2 ms latencies, whereas electromyograms of control animals remained negative (Figure 2).

At 240 days (34 weeks) after lesion, m. quadriceps electromyograms of neurosphere-transplanted pigs were improved up to 2.8–3.1 mV amplitudes with 2.4–2.5 ms latencies, while electromyograms of controls were still negative (Figure 2).

### 3.3. *In Situ* Fate of Grafted Pig Neurosphere Cells

**Figure 3:**
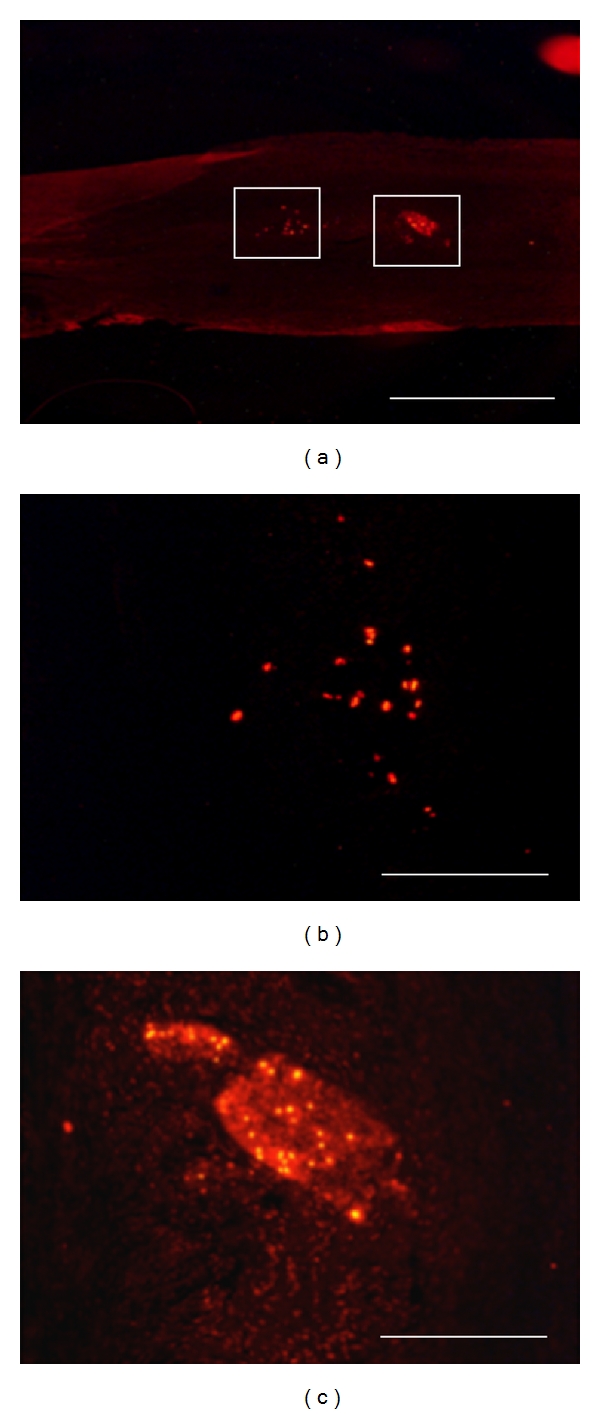
Localization of transplanted BrdU-labeled neurospheres in the bridged nerve at 8 days after lesion, by postmortem immunohistochemistry. BrdU immunoreactivity is revealed by red Alexa-594 fluorescence on longitudinal postfixed graft sections at low (a) and high (b, c) magnifications. Enlarged fields (b, c) are localized as white forms in (a). Transplanted neurosphere cells are found either sparsed (b) or clustered (c) inside the venous bridge. Scale bars: 0.5 mm (a), 100 *μ*m (b, c).

**Figure 4:**
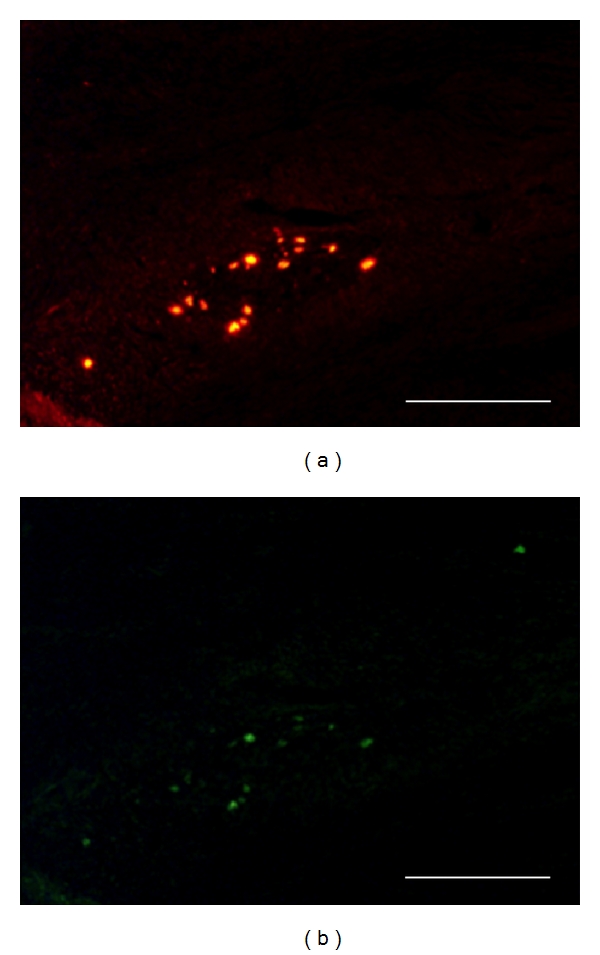
Double BrdU-DCX immunohistochemistry on a sagittal section from a neurosphere-grafted bridged nerve at 8 days after lesion. One given field was sequentially photographed with fluorescent wavelengths revealing, respectively, Alexa-594-labeled BrdU immunoreactivity (a) and Alexa-488-DCX one (b). Note the extensive nuclear colocalization of the immature neuron marker DCX with BrdU (b). Scale bars 100 mm.

**Figure 5:**
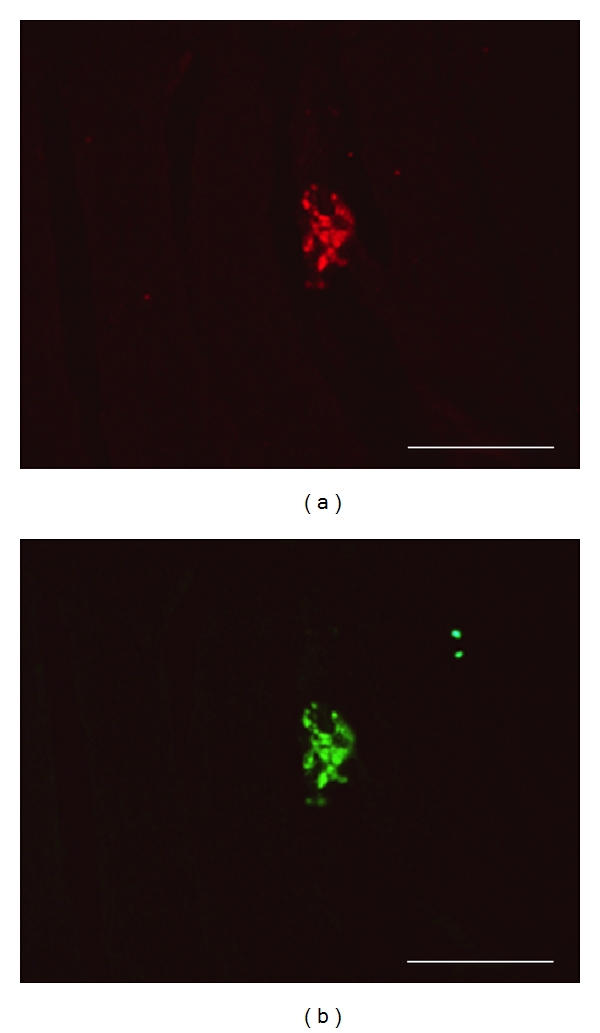
Double BrdU-NeuN immunohistochemistry on a sagittal section from a neurosphere-grafted bridged nerve at 90 days after lesion. One given field was sequentially photographed with fluorescent wavelengths revealing, respectively, Alexa-594-labeled BrdU immunoreactivity (a) and Alexa-488-NeuN one (b). Note the extensive nuclear colocalization of the mature neuron marker NeuN with BrdU (b). Scale bars 100 mm.

In the first experiment, grafted neurosphere cells had been labeled by *in vitro* BrdU incorporation prior to graft. By immunohistochemistry on postmortem venous grafts, groups of BrdU-immunoreactive cells were detected inside the venous tube (Figure 3). In grafts sampled at 8 days after surgery, most of these BrdU+ cells were clustered in sphere-like assemblies (Figures 3(a) and 3(c)) while some were sparsed inside the venous tube (Figure 3(b)). Most of BrdU+ cells were also immunoreactive for doublecortin (DCX) which is a specific marker of immature neurons (Figure 4). At 90 days after surgery, BrdU+ cells were still observed inside the venous tube and displayed immunoreactivity for NeuN, that is, a marker of differentiated neurons (Figure 5).

**Figure 6:**
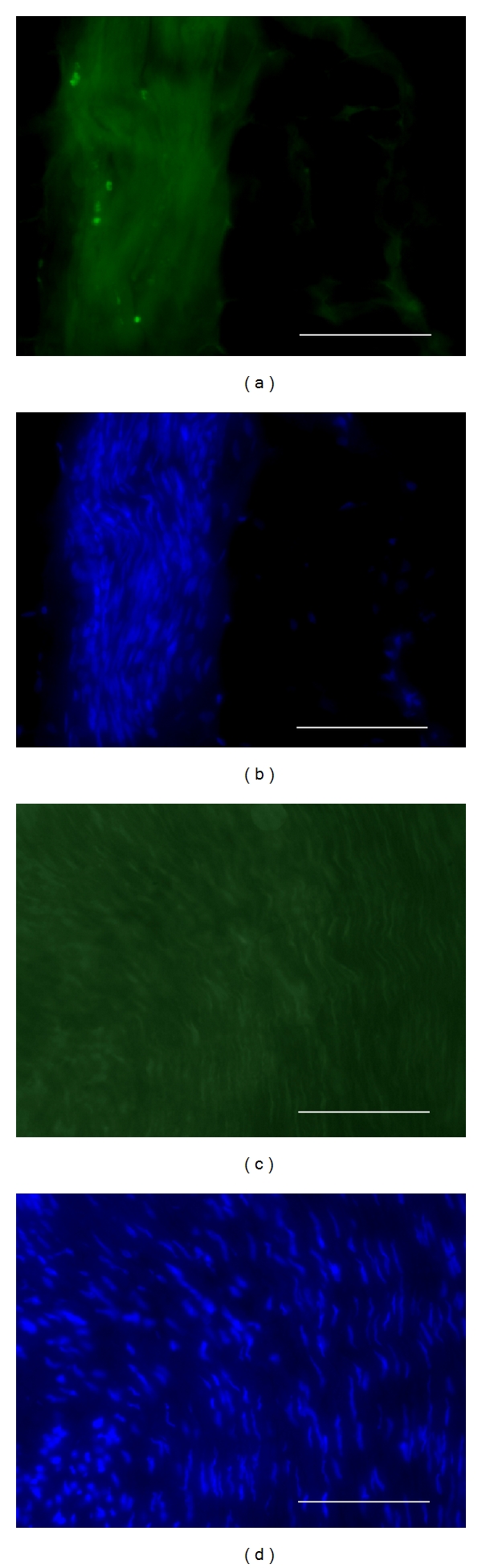
GFP lineage-labelling detection in DAPI-counterstained sagittal sections of venous bridges with (a, b) or without (c, d) transplantation of LV-GFP-labeled neurospheres, at 240 days after lesion. GFP-expressing neurosphere progeny (a, c) and DAPI-labeled tissue cell nuclei (b, d) are detected by fluorescence microscopy by using, respectively, adequate wavelengths. Scale bars: 0.5 mm (a, b), 100 *μ*m (c, d).

**Figure 7:**
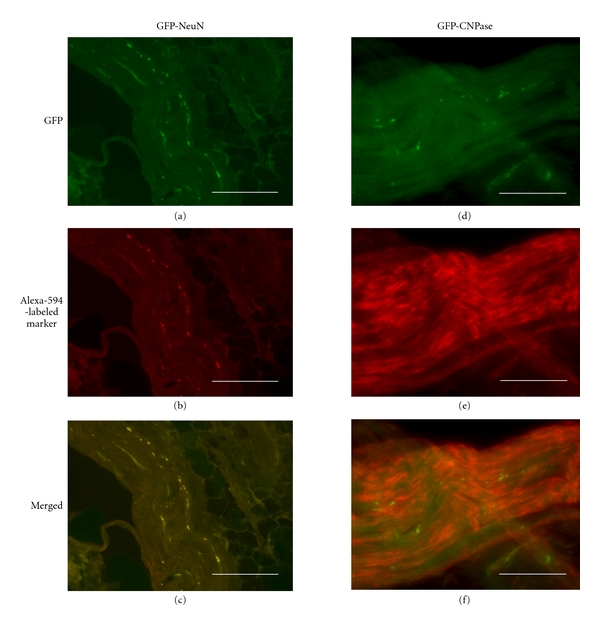
GFP detection and phenotypic marker immunohistofluorescence (NeuN on left column, Schwann cells' CNPase on right column) in sagittal sections of venous bridges which had been transplanted with LV-GFP-labeled neurospheres and were sampled at 240 days after lesion. (a, d) green endogenous fluorescence for detection of lineage-infected neurosphere progeny; (b, e) Alexa-594-revealed phenotypic marker immunoreactivities; (c, f) merged fluorescences. Note yellow-labeled colocalization of GFP-expressing neurosphere progeny with NeuN immunoreactivity (c) but not with CNPase (f). Scale bars: 0.5 mm.

In the second experiment, expanded secondary neurospheres at 8 DIV had been stained by LV-GFP transfection just before collection and postlesional transplantation on the pig nerve gap. All neurospheres displayed fluorescent GFP labeling in most, but not all, cells (Figure 1). At either 45, 90, 180, or 240 days after lesion, sphere transplantation, each vein-bridged and LV-GFP sphere-transplanted nerve segment displayed a number of GFP-fluorescent cells which were exclusively localized inside the nerve fascicles (Figures 6(a) and 6(b)) and absent in nontransplanted control bridges (Figures 6(c) and 6(d)). Combined immunohistofluorescent labeling of either NeuN or neurofilament NF-68 revealed that most of GFP fluorescence was colocalized with these neuronal markers (Figures 7(a)–7(c)). By contrast, GFP fluorescence did not colocalize with either of the glial markers assessed: S-100b (Figures 7(d)–7(f)), GFAP, CNPase. 

**Figure 8:**
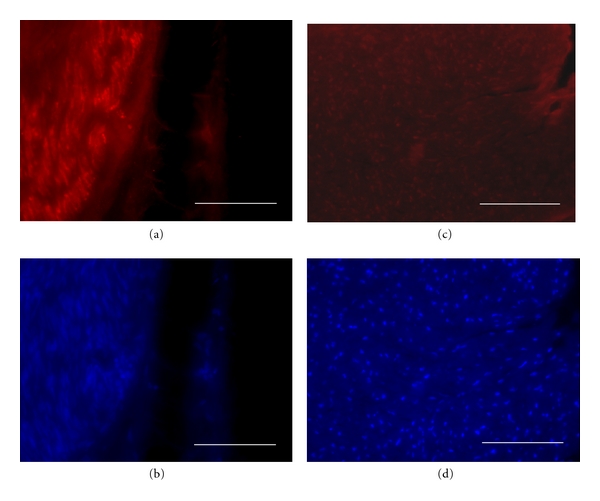
CNPase immunohistofluorescence on sagittal sections of venous bridges without (a, b) or with (c, d) transplantation of LV-GFP-labeled neurospheres, which had been sampled 240 days after lesion. Alexa-594 fluorescence-revealed CNPase immunoreactivity (a, c) is combined with histofluroescent DAPI counterstaining (b, d). Note that Schwann cell labelling density is strikingly higher in neurosphere-transplanted graft (c, d) than in void venous bridge (a, b). Σ*χαλε* *βαρσ*: 100 *μ*m.

Interestingly, at 180 and 240 days after nerve lesion and GFP-expressing neurosphere transplantation, CNPase immunohistofluorescent labeling was much higher than in vein-bridged lesioned controls which had not received neurosphere transplantation (Figure 8). Both the number of CNPase-immunoreactive Schwann cell processes and their labeling intensities were higher in neurosphere-grafted nerves than in controls. By densitometric quantification with the ImageJ software, immunohistochemical CNPase labeling of neurosphere-grafted lesioned nerves, at 240 days after lesion, reached 276 ± 2% of the 100 ± 2.9% value of lesioned controls which were devoid of neurospheres; this difference being statistically significant at *P* < 0.001 by Anova.

## 4. Discussion

Degeneration of the distal axon segment is the consequence of axotomy and the challenge of nerve repair is to connect the proximal stump to this distal segment. For long nerve gap, it is a race between axon regeneration and distal degeneration with phenotypic change of Schwann cells [24]. Autologous nerve graft is the gold standard but results are not satisfactory [15]. Any alternative is currently believed to require a neuroguide, providing a permissive microenvironment to increase axonal regeneration and to preserve supportive cells and distal axons. Stem cells have already been studied but differentiation was mostly towards supportive cells. Neural stem cells have been reported to become integrated and provide new neurons after transplantation within central nervous system with permissive microenvironment [25]. In order to test the efficiency of this type of graft for postlesional repair of peripheral nerves, we have established a primary culture of adult pig neural stem cells which generate neurons *in vitro* in our previous report [27]. In the present study, we show for the first time in adult pig that transplantation of *in vitro* preexpanded adult neural stem cells into a peripheral nerve gap inside a venous bridge both leads to genesis of longitudinally aligned neurons and improves functional recovery.

### 4.1. Methodological Considerations

The present experimental protocol of nerve lesion: transsection and 30 mm long nerve substance removal, has been chosen to mimic the common cases of long nerve injury in human clinics. In these cases indeed, whatever be the initial type of injury (section, tearing, crush), the surgical regularization of both nerve stumps corresponds to a model of transsection.

Autologous vein segment graft as a nerve conduit has been established as an effective treatment for the repair of nerve gaps less than 3 cm [29, 30]; it is used in hand surgery [31] and in pediatrics [32]. In a recent study [33], histomorphological examination of the sections proximal to, from, and distal to the repair zone over three months revealed less epineural scarring, a thinner epineurium, more regenerated axons and fewer inflammatory cells in groups where vein grafting was used, because the vein graft provided additional mechanical and chemical support in the size discrepancy of the nerve regeneration. A small vein graft keeps its property for end-to-side neurorrhaphy [34], but size discrepancy between the donor and recipient nerves and length of the bypass are another problem with the risk of collapse. Vein bridging also gives excellent results in secondary repair of neglected injuries [35]. The present protocol is routinely realized in adult pig, as a convenient training system for human-operating surgeons.

The present postlesional cell therapy attempt used an *in vitro* expanded primary culture of adult pig brain neurospheres which had been extensively characterized in a previous study [27]. We had indeed shown that the present protocols of adult pig subventricular zone sampling and neurosphere assay allow to purify and maintain true neural stem cells with unlimited proliferative potential, self-renewal capacity, and ability to generate all neural cell types [27]. In particular, these neurospheres from adult pig SVZ displayed the same proportions of neurons (25%), astrocytes (70%), and oligodendrocytes (5%) as neurospheres from various adult rodent brain structures [36–38].

The procedure for fluorescent lineage labeling of grafted neurospheres used a lentiviral vector of GFP which has been validated previously [28]. Freshly prepared lentiviral particles were quantified by multiplicity of infection (MOI) determination using routine virologist assays, for optimization of the neurosphere labeling. We checked that this *in vitro* procedure allowed extensive labeling of adult pig neurospheres without alteration of their cell dynamics. It allowed subsequent identification of grafted cell progenies *in situ* merely by histofluorescence, which easily enabled to perform phenotype characterization by immunohistochemistry as revealed with different fluorophores.

### 4.2. Functional Recovery Enhancement by Vein-Guided Neurosphere Transplantation

Electromyographic evaluation of the muscle which is innervated by the lesioned nerve demonstrated that our protocol of venous-graft-assisted neurosphere transplantation strongly improved functional recovery at 6 months after lesion, as compared to controls. Our subsequent histofluorescent analyses provided putative cytological substrates for such positive influence of grafted ANSC-derived cells.

In the same time, clinical evaluation was based on a simple observation. The section of the crural nerve pulled a deficient flexion of the thigh on the pond. The only recoveries of normal step were observed on the transplanted animals. The main question was to know if this recovery was bound to accelerated axonal regrowth or to the creation of a functional intermediate neuronal bypass. In fact we have a double observation of neurons and regrowth of the axons of the host.

### 4.3. Neuronal Outcome of Grafted Neurospheres inside the Venous Bridge

In the present cell therapy attempt, the grafted neurospheres yielded exclusively neuronal progenies whatever be the postlesional delay. This outcome is striking since the neuronal yields from adult SVZ neural stem cells are known to reach only 25% neurons *in vitro* and 75% in the olfactory pathway *in vivo* [25, 37, 39]. The present *in vivo* result indicates that the venous graft provided an efficient proneurogenic environment for the transplanted neurospheres. It is in keeping with the recent demonstrations that vascular walls favor neurogenesis from adult neural stem cells [38, 40, 41], which is mediated in some rodent models by the vascular endothelium-derived growth factor (VEGF) [42]. Our results suggest that choosing a venous trunk versus an artificial neuroguide to bridge a nerve gap is an interesting solution because of intrinsic property. Furthermore, all surgeons are able to take a vein trunk on superficial vein network and this is much less expensive than neurotubes in emergency conditions. At the same time, to understand mechanism of interaction between endothelial cells and grafted neural stem cells will allow progress for the development of new artificial neurotubes. More generally, local tissue microenvironment has been formally demonstrated to be determinant on the phenotypic fate of stem-cell-derived progenies: for instance, nonneurogenic neural stem cells from the adult spinal cord generated neurons exclusively, once transplanted into the hippocampus [43].

These newly formed neurons survived at long delays after the original transplantation, which indicates they have been successfully integrated inside the severed nerve. Further, the linear distribution of these neuronal perikarya, parallel to the longitudinal axis of the lesioned nerve, suggests that the new neurons might be interconnected into multisynaptic nets bridging the lesional gap of preexisting nerve fibers. This multisynaptic nets have been described after neural stem cells graft [44, 45] This interpretation is also supported by the evolution of transplanted cell clusters across time inside the venous graft, that is, from spherical assemblies in the first week after lesion to linear chainlike successions of sparse cells at longer delays. Such outcome arises apart of the strategies for peripheral nerve repair which have been worked out thus far and consist in stimulating either axonal regrowth into the distal nerve end or remyelination with additional Schwann cells [24].

The present results contrast with previous ones using different kinds of transplanted cells, like fœtal neural stem cells, which triggered Schwann cell like proliferation in the lesioned peripheral nerve [46], promoting in turn myelinisation and indirectly regeneration [47]. In addition, transplantation of immortalized C17.2 cells from postnatal cerebellum, which were shown to be distinct of neural stem cells [48], has been reported to trigger tumor formation in a rat model of injured sciatic nerve [49]. Adult neural stem cells are especially relevant for cell therapy from this viewpoint, because they were demonstrated to display exceptionally high resistance to tumorigenesis and senescence [50].

### 4.4. Possibility to Graft New Neurons into a Peripheral Nerve Gap

There are two approaches, either to transplant neurons, or to transplant stem cells possessing neuronal differentiation potential. For primary neurons, dorsal root neurons can be prepared *in vitro* and their axons stretch-growth [20]. Moreover, engineered nervous tissue construct consisted of longitudinally aligned axonal tracts spanning two neuronal populations, embedded in a collagen matrix and inserted in a PGA tube [44] demonstrated neuronal survival during 112 days after surgical repair of the sciatic nerve in the rat. But only studies on the big animals allow the study of nerve gaps in good conditions.

### 4.5. High Survival Rate of Allogenic Transplants

In our study, adult neural stem cells were prepared *in vitro* after explantation of SVZ cells from another pig, and allogenic grafted cells were integrated without immunosuppressive therapy. The same observation was noted for embryonic rat progenitors for the adult rat sciatic nerve [44] and with xenograft of embryonic rat neural stem cells into a collagen conduit for the rabbit facial nerve injury model [51]. In these attempts, grafted cells were expanded *in vitro* before transplantation, like in the present study.

### 4.6. Activation of Intrinsic Schwann Cells by Neurosphere Transplantation

Phenotypic characterization of grafted cell progenies showed that our transplanted neurospheres generated no Schwann cell directly, but they triggered increases of the number and activity of this cell population as compared to controls. CNPase+ cells were observed along axonal regeneration but were always LV-GFP-. This result is in keeping with a previous report showing that venous graft favored Schwann cells proliferation after nerve lesion [52]. However, since in the present study controls have received a neurosphere-devoid venous graft, our results indicated that neurosphere cells emit diffusible signals that stimulate Schwann cells. Neurospheres have indeed been shown *in vitro* to secrete neurotrophic factors [53, 54].

## 5. Conclusion

The positive impact of the present cell therapy protocol can be attributed to genesis and integration of new neurons inside the lesioned nerve and/or to indirect activation of intrinsic Schwann cell population. However, Schwann cell activation was previously shown to occur after grafting a venous segment onto the lesioned nerve without neural stem cells inside. Therefore, the specific benefit of the present procedure is likely caused by the neurosphere transplantation, and hence by the genesis of new neurons inside the lesioned nerve. LV-GFP is confirmed to provide an excellent marker for grafted cells across long-time studies. Both new neuron genesis and host Schwann cell proliferation contributed to regenerate new axon fascicles in the host to bridge the long nerve gap, and the functionality of this histological repair was demonstrated in the same animals by electromyography and clinics. Large animals, and especially the pig, allow repair and study of long nerve gap with clinical conditions close to human. These results were obtained without adjunction of extracellular matrix or extrinsic growth factor. The present study thus provides a hope for improvement in human clinics.

## Figures and Tables

**Table 1:** List of primary antibodies used.

Antigen	Antibody	Supplier	Cell-type specificity	Dilution
BrdU	Rat polyclonal	ImmunologicalDirect.com	none	1/100
CNPase	Mouse monoclonal	Sigma, L'Isle-d'Abeau, France; C-5922, clone 11-5b	Schwann cells	1/200
GFAP	Rabbit polyclonal	Dako, Trappes, France; Z0334	Astrocytes, neural stem cells	1/1000
NeuN	Mouse monoclonal	Chemicon, MAB377	Mature neurons	1/100
NF-68	Mouse monoclonal	BD-Biosciences, Heidelberg, Germany	Mature neurons	1/400
S100*β*	Rabbit polyclonal	Dako, Trappes, France	Astrocytes, Schwann cells	1/500
